# Acetylation of Lactate Dehydrogenase Negatively Regulates the Acidogenicity of Streptococcus mutans

**DOI:** 10.1128/mbio.02013-22

**Published:** 2022-08-31

**Authors:** Qizhao Ma, Yangyang Pan, Yang Chen, Shuxing Yu, Jun Huang, Yaqi Liu, Tao Gong, Qiong Zhang, Qun Sun, Jing Zou, Yuqing Li

**Affiliations:** a State Key Laboratory of Oral Diseases, National Clinical Research Center for Oral Diseases, West China Hospital of Stomatology, Sichuan University, Chengdu, China; b Department of Pediatric Dentistry, West China Hospital of Stomatology, Sichuan University, Chengdu, China; c Department of Pediatric Dentistry, School and Hospital of Stomatology, Wenzhou Medical University, Wenzhou, China; d Key Laboratory of Bio-resources & Eco-environment of the Ministry of Education, College of Life Sciences, Sichuan University, Chengdu, China; University of Geneva

**Keywords:** *Streptococcus mutans*, lysine acetylation, acetyltransferase, lactate dehydrogenase

## Abstract

Lysine acetylation, a ubiquitous and dynamic regulatory posttranslational modification (PTM), affects hundreds of proteins across all domains of life. In bacteria, lysine acetylation can be found in many essential pathways, and it is also crucial for bacterial virulence. However, the biological significance of lysine acetylation events to bacterial virulence factors remains poorly characterized. In Streptococcus mutans, the acetylome profiles help identify several lysine acetylation sites of lactate dehydrogenase (LDH), which catalyzes the conversion of pyruvate to lactic acid, causing the deterioration of teeth. We investigated the regulatory mechanism of LDH acetylation and characterized the effect of LDH acetylation on its function. We overexpressed the 15 Gcn5 *N*-acetyltransferases (GNAT) family members in S. mutans and showed that the acetyltransferase ActA impaired its acidogenicity by acetylating LDH. Additionally, enzymatic acetyltransferase reactions demonstrated that purified ActA could acetylate LDH *in vitro*, and 10 potential lysine acetylation sites of LDH were identified by mass spectrometry, 70% of which were also detected *in vivo*. We further demonstrated that the lysine acetylation of LDH inhibited its enzymatic activity, and a subsequent rat caries model showed that ActA impaired the cariogenicity of S. mutans. Collectively, we demonstrated that ActA, the first identified and characterized acetyltransferase in S. mutans, acetylated the LDH enzymatically and inhibited its enzymatic activity, thereby providing a starting point for the further analysis of the biological significance of lysine acetylation in the virulence of S. mutans.

## INTRODUCTION

Dental caries result from the dissolution of the tooth mineral structure by the acids formed by tooth-dwelling bacteria through their metabolism of dietary carbohydrates (1, 2). As the significant etiologic agent of dental caries, Streptococcus mutans possesses many physiological characteristics that are relevant to the initiation and progression of dental caries (3). Via the rapid fermentation of several dietary carbohydrates, S. mutans can generate organic acids (acidogenicity), primarily lactic acid, catalyzed by lactate dehydrogenase (LDH, encoded by *ldh*). LDH is an oxidoreductase isoenzyme that catalyzes the reversible reaction between pyruvate and lactic acid. LDH and lactic acid are direct causative factors for the demineralization of teeth and also impact other caries-related microbial flora during the cariogenic process (4).

The acidogenicity of S. mutans will be suicidal if it cannot tolerate the acid. Therefore, S. mutans has developed an adaptive acid tolerance response (ATR) by which to survive this acidic stress and further use carbohydrates for metabolic activities in this changing acidic environment (aciduricity) (5). The aciduricity of S. mutans depends on a complex regulatory mechanism to maintain the cytoplasm at a physiological pH. For example, when exposed to mildly acidic conditions, S. mutans survives better during subsequent low pH challenges (6, 7). In addition, acidogenicity and aciduricity allow S. mutans to dominate other microbial flora within the dental biofilm, which creates a cariogenic environment.

Protein lysine acetylation is a prevalent and dynamic posttranscriptional modification (PTM) that regulates protein structure and function (8, 9). Lysine acetylation modifies existing proteins by transferring an acetyl group from a donor metabolite to a lysine residue of the target substrate instead of translating new proteins, enabling bacteria to quickly adapt to a changing environment (10, 11). Protein lysine acetylation can be achieved by two distinct mechanisms: nonenzymatic acetylation (chemical acetylation), in which acetyl phosphate (AcP) or acetyl-coenzyme A (Ac-CoA) serves as a donor of the acetyl group, and enzymatic acetylation, which relies on lysine acetyltransferases (KATs) to transfer the acetyl group from Ac-CoA (12–14). Notably, increased global analyses of lysine acetylation have been reported in bacteria, including Escherichia coli, Mycobacterium tuberculosis, Porphyromonas gingivalis, Bacillus subtilis, Pseudomonas aeruginosa, and Salmonella enterica (15–21). This has laid the foundation for understanding the effect of lysine acetylation on diverse cellular processes, such as central metabolism, protein translation, and bacterial virulence and survival.

Previously, we found that several lysine sites of LDH could be acetylated in the global acetylation profiles of S. mutans (22). However, the regulatory mechanisms and biological effects of LDH acetylation are still unknown. KATs identified in bacteria to date belong to the yeast Gcn5 *N*-acetyltransferases (GNATs) (23, 24). GNATs acetylate a broad range of substrates, including amino acids, peptides, nucleotides, and proteins. The S. mutans UA159 genome contains 15 genes whose products are annotated as GNATs, the functions of which are yet to be defined. Therefore, we investigated whether these uncharacterized GNATs regulate the acetylation of LDH in S. mutans.

Here, we found that ActA increases the acetylation of LDH by using constructed S. mutans strains that overexpressed the 15 GNAT superfamily genes. The acetylated sites of LDH have been identified by mass spectrometric analysis. In addition, the increased acetylation of LDH reduced its enzymatic activity, leading to decreased lactic acid production. Our work revealed an important regulatory function of lysine acetylation in the acidogenicity of S. mutans.

## RESULTS

### Identification of putative uncharacterized KAT.

Our previous acetylome analysis showed that several lysine sites of LDH are acetylated in S. mutans (Table S1). There are 15 putative GNATs in S. mutans. To date, the known KATs are highly specific (25); therefore, we sought the regulatory mechanism behind LDH acetylation, hypothesizing that the acetylated lysine sites of LDH could be attributed to these uncharacterized KATs.

10.1128/mbio.02013-22.6 TABLE S1 Identified lysine acetylation sites of LDH in the acetylome profiles of S. mutans. Download Table S1, DOCX file, 0.1 MB. Copyright © 2022 Ma et al. 2022 Ma et al. https://creativecommons.org/licenses/by/4.0/ This content is distributed under the terms of the Creative Commons Attribution 4.0 International license.

We overexpressed each of the GNATs in S. mutans UA159 to examine whether LDH is acetylated by these uncharacterized KATs *in vivo*. As the LDH catalyzes the conversion of pyruvate to lactic acid, we compared the differences in the acidogenicity of these strains by monitoring the decrease in the pH of the supernatant in a glycolytic pH drop assay. The results showed that the pH of S. mutans overexpressing *actA* decreased gradually over 60 min versus that of a control, and no significant pH differences were observed in other overexpressed strains (Fig. S1). Therefore, we selected ActA for a further assessment of its ability to regulate the acidogenicity of S. mutans.

10.1128/mbio.02013-22.1 FIG S1 Effect of the overexpression of GNAT family members on the acidogenicity of S. mutans. The pH values of the supernatant of S. mutans overexpressing 15 GNAT family member (*actA-actH* [A] and *actI-actO* [B]) cultures grown in the presence of 1% glucose over 60 min. Download FIG S1, TIF file, 0.2 MB. Copyright © 2022 Ma et al. 2022 Ma et al. https://creativecommons.org/licenses/by/4.0/ This content is distributed under the terms of the Creative Commons Attribution 4.0 International license.

### ActA inhibited the acidogenicity of S. mutans.

An *actA* in-frame markerless deletion strain was constructed to evaluate the function of ActA. First, we measured the mRNA level of *actA* in S. mutans UA159, UA159/pDL278, UA159/pDL278*-actA*, and UA159 Δ*actA* strains, using quantitative real-time polymerase chain reaction (qRT-PCR). As shown in Fig. S2, the expression level of *actA* in the strain overexpressing *actA* increased significantly, about 260-fold, compared with that of UA159/pDL278. No *actA* transcripts were detected in the *actA* deletion strain, as expected. These results indicate that both the *actA* in-frame markerless deletion strain and the overexpression strain were successfully constructed.

10.1128/mbio.02013-22.2 FIG S2 Gene expression level of *actA* in the indicated strains. The gene expression level of *actA* was determined by quantitative RT-PCR and was calculated using the 2-ΔΔCt method with values normalized to the reference gene 16s rRNA. The results are presented as mean ± SD (****, *P* < 0.0001). Download FIG S2, TIF file, 0.1 MB. Copyright © 2022 Ma et al. 2022 Ma et al. https://creativecommons.org/licenses/by/4.0/ This content is distributed under the terms of the Creative Commons Attribution 4.0 International license.

**FIG 1:**
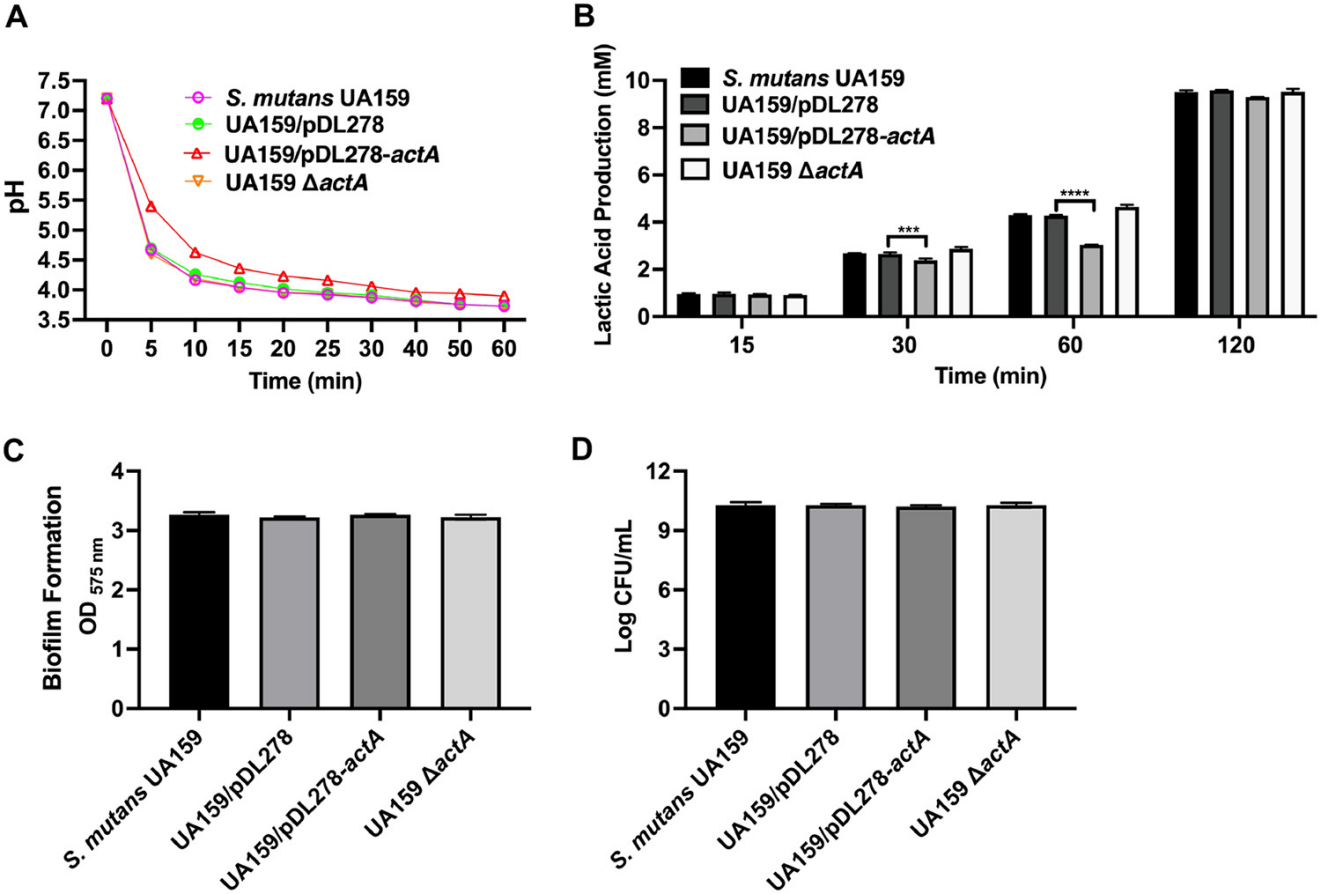
Effect of *actA* on the acidogenicity of S. mutans. (A) The pH value of the supernatant of S. mutans UA159, UA159/pDL278, UA159/pDL278*-actA*, and UA159 Δ*actA* cultures grown in the presence of 1% glucose over 60 min. (B, C, and D) After the strains were cultured in BHIS (1% sucrose wt/vol) under anaerobic conditions for 24 h, the lactic acid production was measured by a lactic acid assay kit, following the manufacturer’s protocol (B), the biofilm biomass was determined by a crystal violet staining assay (C), and the colony forming units (CFU) were counted (D). The results are presented as mean ± standard deviation (SD) (**, *P* < 0.01; ***, *P* < 0.001; and ****, *P* < 0.001).

In the glycolytic pH drop assay, no significant pH differences were observed in the supernatant of the UA159 Δ*actA* strain over 60 min compared with the parental strain (Fig. 1A). The lactic acid productions of different strains were measured in the presence of glucose as the sole carbohydrate source. At 30 and 60 min, the lactic acid production of the biofilm decreased in the strain overexpressing *actA*, with no significant differences in the UA159 Δ*actA* strain, compared with UA159/pDL278 and S. mutans UA159, respectively (Fig. 1B).

The biofilm biomass and colony forming units (CFU) were measured to exclude the possibility of a decrease in lactic acid production caused by different total bacterial counts. As shown in Fig. 1C and D, no significant differences were observed in the biofilm biomass or CFU between the S. mutans UA159, UA159/pDL278, UA159/pDL278*-actA*, and UA159 Δ*actA* strains. These findings showed that *actA* impaired the acidogenicity of S. mutans, whether in the biofilm or planktonic condition.

### ActA inhibited the aciduricity of S. mutans.

**FIG 2:**
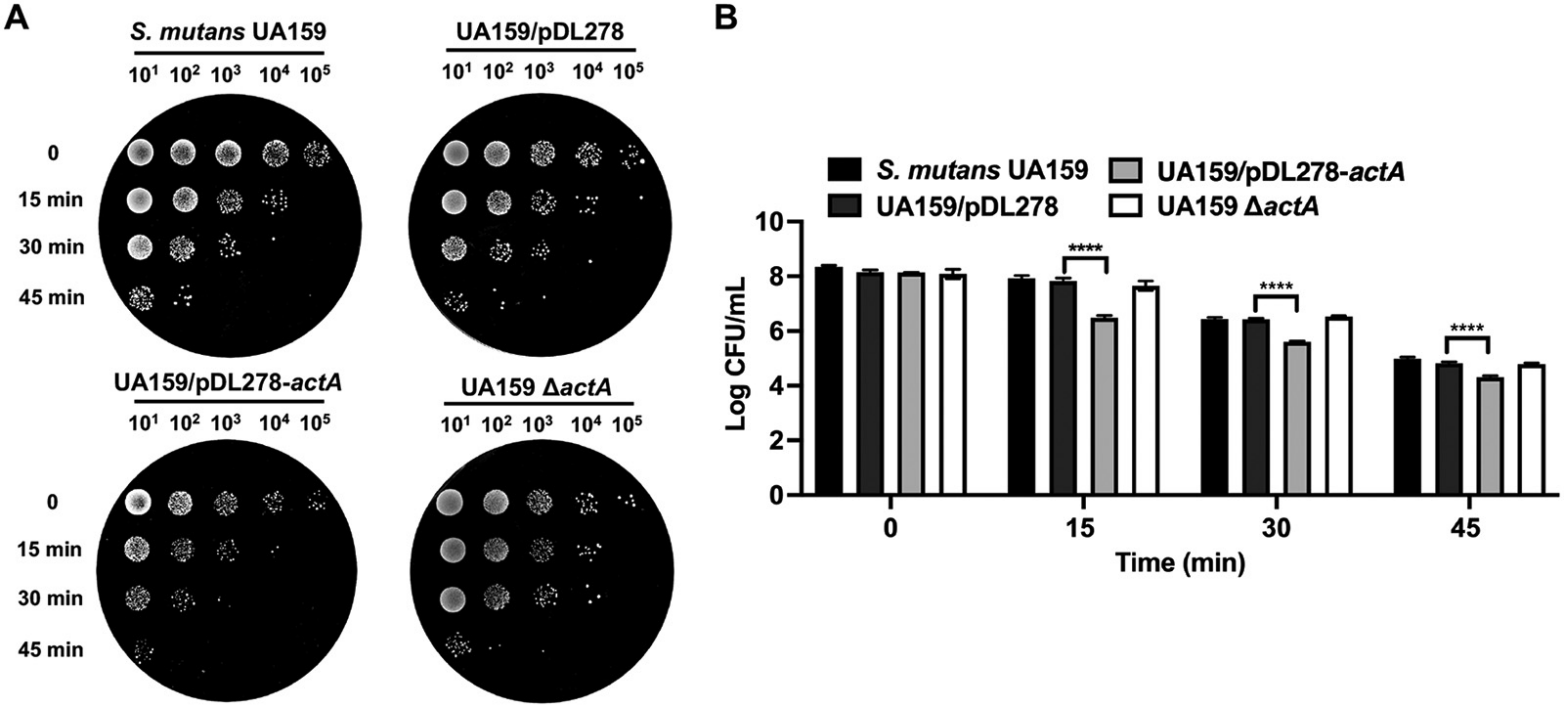
Effect of *actA* on the aciduricity of S. mutans. (A and B) S. mutans UA159, UA159/pDL278, UA159/pDL278*-actA*, and UA159 Δ*actA* strains exposed to a killing pH of 2.8 for 15, 30, and 45 min, respectively. The survivors were recovered by culturing on BHI-agar plates and incubating anaerobically for 48 h at 37°C. Representative images are shown from three independent experiments (A). The concentrations of bacterial cells were calculated by CFU (B). The results are presented as mean ± SD (****, *P* < 0.0001).

Another virulence factor of S. mutans is aciduricity, that is, the ability to grow and survive in a low-pH environment. We used an ATR assay to assess the acid stress-mediated killing of the S. mutans UA159, UA159/pDL278, UA159/pDL278*-actA*, and UA159 Δ*actA* strains by exposing cells grown at a pH of 7.2 to a killing pH of 2.8 without pre-exposure to an adapted pH (5.5). As judged by the decreased percentage of surviving CFU, it was evident that the strain overexpressing *actA* exhibited an impaired ability to survive in the lethal pH, with no significant differences for the UA159 Δ*actA* strains, compared with UA159/pDL278 and S. mutans UA159, respectively (Fig. 2A and B). The acid sensitivity of the strain overexpressing *actA* suggested that ActA affected the acid tolerance of S. mutans.

### Identification of putative ActA substrate proteins by mass spectrometry.

**FIG 3:**
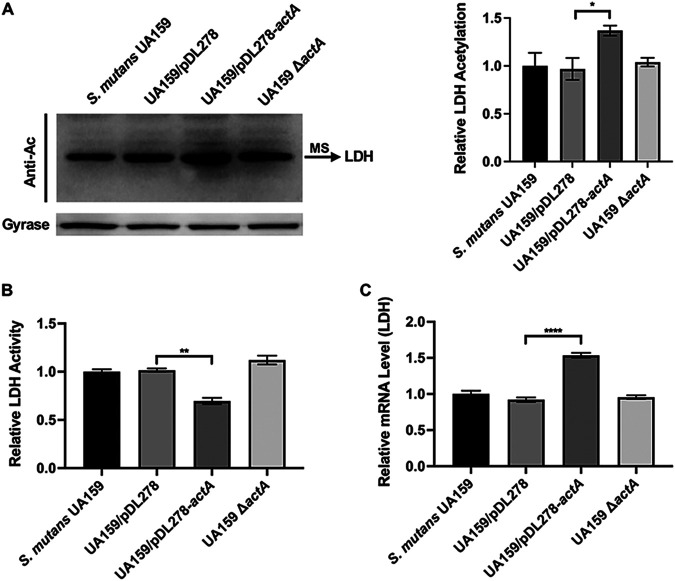
Effect of *actA* on total protein acetylation, LDH activity, and *ldh* transcription. (A) The extracted total proteins were analyzed by anti-acetyl lysine Western blotting. The upregulated acetylated protein was identified by mass spectrometry (MS). The band signals were quantified with ImageJ software and normalized to the control. (B) The LDH activity assay was performed on S. mutans UA159, UA159/pDL278, UA159/pDL278*-actA*, and UA159 Δ*actA* strains, following the manufacturer’s protocol. (C) The gene expression level of *actA* in the indicated strains was determined by quantitative real-time polymerase chain reaction (RT-PCR) and calculated using the 2^-ΔΔCt^ method with values normalized to the reference gene 16S rRNA. The results are presented as mean ± SD (*, *P* < 0.05; **, *P* < 0.01; and ****, *P* < 0.0001).

We compared the acetylation profiles of the S. mutans UA159, UA159/pDL278, UA159/pDL278*-actA*, and UA159 Δ*actA* strains via anti-acetyl lysine Western blotting to determine the substrates of ActA, given the evidence that ActA functions as a KAT. In the acetylated proteins’ profile, we observed an upregulated acetylation band in the strain overexpressing *actA* compared with other strains (Fig. 3A and Fig. S3). We used mass spectrometry (MS) for unbiased identification and found that the upregulated acetylation band was LDH (Table S2).

10.1128/mbio.02013-22.3 FIG S3 Effect of ActA on the protein expression levels of S. mutans. The extracted total proteins were analyzed by Coomassie blue-stained SDS-polyacrylamide gel electrophoresis to ensure equivalent loading and to detect the protein expression level. Download FIG S3, TIF file, 0.2 MB. Copyright © 2022 Ma et al. 2022 Ma et al. https://creativecommons.org/licenses/by/4.0/ This content is distributed under the terms of the Creative Commons Attribution 4.0 International license.

10.1128/mbio.02013-22.7 TABLE S2 Identified target substrate (LDH) of ActA by MS analysis. Download Table S2, DOCX file, 0.1 MB. Copyright © 2022 Ma et al. 2022 Ma et al. https://creativecommons.org/licenses/by/4.0/ This content is distributed under the terms of the Creative Commons Attribution 4.0 International license.

As the lysine acetylation of proteins could regulate enzymatic functions, we tested the effect of ActA on LDH activity. The results showed that the LDH activity of the strain overexpressing *actA* decreased significantly, with no significant differences in LDH activity in the UA159 Δ*actA* strains compared with UA159/pDL278 and S. mutans UA159, respectively (Fig. 3A and B). Meanwhile, we measured the mRNA level of *ldh* in the S. mutans UA159, UA159/pDL278, UA159/pDL278*-actA*, and UA159 Δ*actA* strains using qRT-PCR. As shown in Fig. 3C, the expression level of *ldh* in the strain overexpressing *actA* increased significantly, with no significant differences in *ldh* expression in the UA159 Δ*actA* strains, compared with UA159/pDL278 and S. mutans UA159, respectively. These findings indicated that ActA might directly acetylate LDH, which in turn decreases LDH activity.

### ActA acetylates LDH via an enzymatic mechanism.

**FIG 4:**
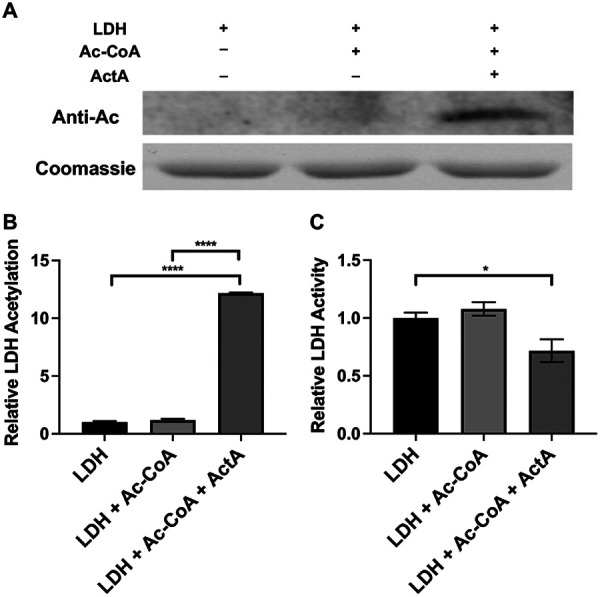
Effect of ActA on LDH acetylation and activity. Coomassie staining (A), anti-acetyl lysine Western blotting (A), and an LDH activity assay (C) of LDH were incubated with ActA as the acetyltransferase and Ac-CoA as the acetyl donor for 3 h at 37°C. The anti-acetyl lysine band signals were quantified with ImageJ software and normalized to the control (B). The results are presented as mean ± SD (*, *P* < 0.05 and ****, *P* < 0.0001).

The ActA and LDH were cloned into expression vectors and purified from Escherichia coli to investigate whether ActA acted as LDH KAT. Furthermore, we selected another KAT ActG in S. mutans, reported in our previous study, as a control (26). Specifically, ActG, one member of the GNAT family in S. mutans, regulates biofilm formation by acetylating glucosyltransferases (Gtfs). Therefore, we selected ActG as a control by which to detect the effect of ActA on acetylating LDH. The *in vitro* acetylation assays of LDH were carried out in the presence or absence of Ac-CoA and/or ActA or ActG. As shown in Fig. 4A and Fig. S4, ActA could only acetylate LDH in the presence of Ac-CoA, Ac-CoA could not acetylate LDH in the absence of KAT ActA, and ActG could not acetylate LDH, even in the presence of Ac-CoA.

10.1128/mbio.02013-22.4 FIG S4 Effect of ActA and ActG on LDH acetylation. LDH was incubated with ActA or ActG as the acetyltransferase and Ac-CoA as the acetyl donor for 3h at 37°C. Then, LDH was analyzed by anti-acetyl lysine Western blotting. Download FIG S4, TIF file, 0.1 MB. Copyright © 2022 Ma et al. 2022 Ma et al. https://creativecommons.org/licenses/by/4.0/ This content is distributed under the terms of the Creative Commons Attribution 4.0 International license.

LDH was incubated with Ac-CoA in the presence or absence of ActA and then subjected to mass spectrometry (MS) to identify the specific lysine residues of LDH acetylated by ActA *in vitro*. The LDH incubated with Ac-CoA was not acetylated. The LDH incubated with ActA and Ac-CoA was acetylated on 10 sites, as determined by MS/MS (Table S3), the majority (70%) of which were also detected *in vivo* (Table S1).

10.1128/mbio.02013-22.8 TABLE S3 Identified lysine acetylation sites of LDH by MS/MS fragmentation *in vitro* acetylation analysis. Download Table S3, DOCX file, 0.1 MB. Copyright © 2022 Ma et al. 2022 Ma et al. https://creativecommons.org/licenses/by/4.0/ This content is distributed under the terms of the Creative Commons Attribution 4.0 International license.

In addition, we investigated the effect of the lysine acetylation of LDH on its enzymatic activity. As shown in Fig. 4C, the acetylated LDH negatively regulated its enzymatic activity. Altogether, these findings showed that ActA is a KAT in S. mutans that directly acetylates LDH and negatively regulates its enzymatic activity.

### *actA* overexpression impaired the cariogenicity of S. mutans.

**FIG 5:**
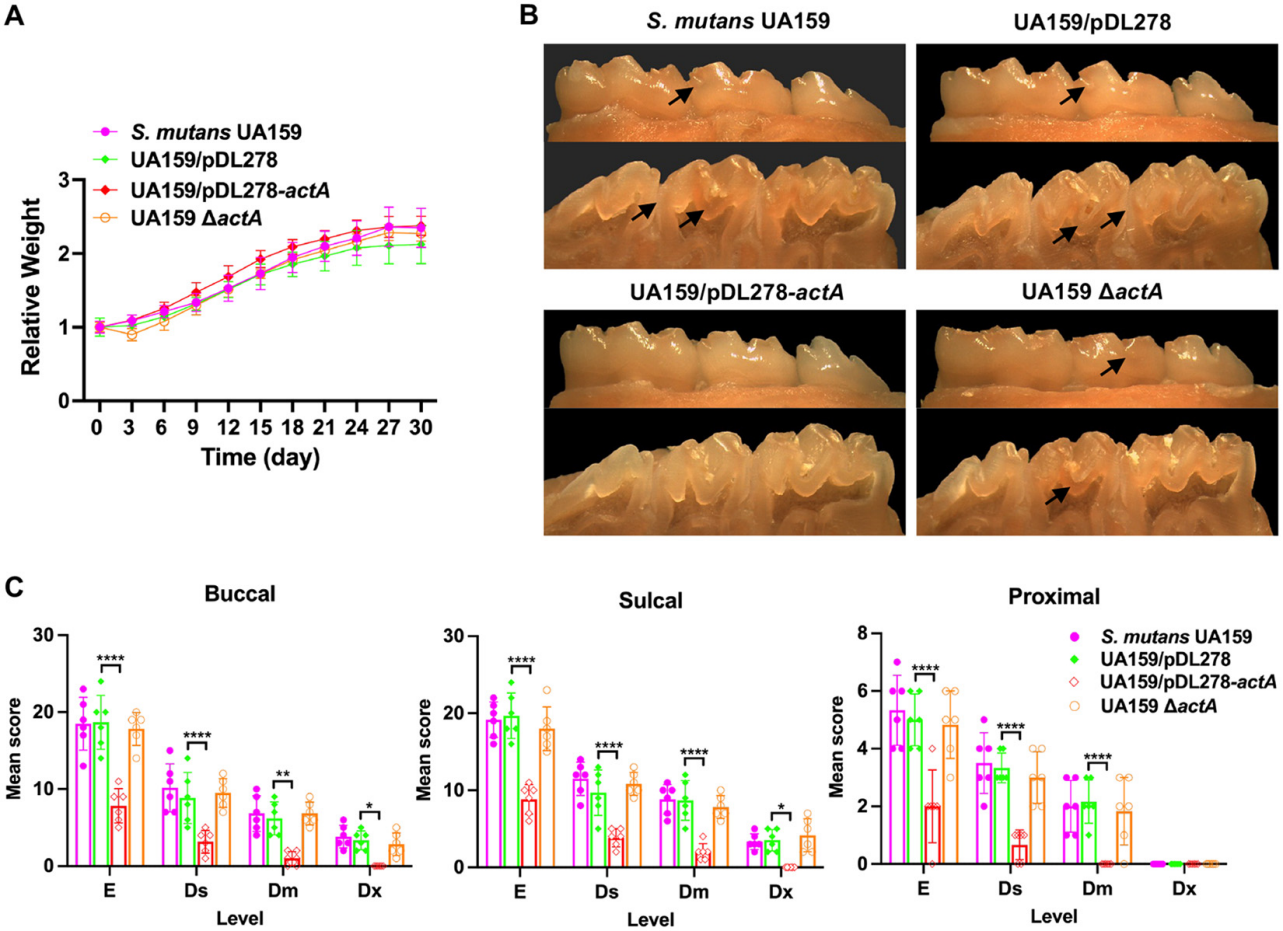
Effect of *actA* on the cariogenicity of S. mutans. (A) The rats were weighed every 3 days until the termination of the experiment. (B) Representative images of the buccal (above panel) and sulcal (below panel) of the mandible with molars are displayed. The arrows indicate the representative carious lesions. (C) A statistical chart of the Keyes’score is shown in Table 1. Each plot represents the caries score of each rat (*n* = 6) on the buccal, sulcal, and proximal surfaces, respectively. The severity of carious lesions was classified into enamel only (E), slight dentinal (Ds), moderate dentinal (Dm), and extensive dentinal (Dx). The results are presented as mean ± SD (*, *P* < 0.05; **, *P* < 0.01; and ****, *P* < 0.0001).

The strain overexpressing *actA* regulated the acetylation of LDH and reduced its enzymatic activity, decreasing lactic acid production in S. mutans. A well-established rat caries model was employed with four groups receiving S. mutans UA159, UA159/pDL278, UA159/pDL278*-actA*, or UA159 Δ*actA* strains to investigate whether the overexpression of *actA* affects the cariogenicity of S. mutans. During the entire study, no significant differences were observed in the pattern or amount of meals consumed by the rats in different groups. Consistent with this, no significant differences were detected in the weight gains of rats between different groups (Fig. 5A). After the rats were sacrificed, oral plaque samples were collected by sonicating molars in sterile PBS. The strains of UA159/pDL278 and UA159/pDL278*-actA* were verified by PCR using universal primers (Fig. S5A and Table S5). In addition, the bacterial load of S. mutans in each group was calculated from the plaque sample. As shown in Fig. S5B, there were no significant differences in the loads of UA159/pDL278*-actA* and UA159 Δ*actA* compared with those of UA159/pDL278 and S. mutans UA159, respectively.

10.1128/mbio.02013-22.5 FIG S5 Effect of *actA* on the bacterial load of S. mutans in the rat caries model. Oral plaque samples were collected when all rats were sacrificed. The samples were divided into two parts, one part for verifying the strains of UA159/pDL278 and UA159/pDL278-*actA* by PCR (A) and the other part for counting the bacterial load by CFU (B). Download FIG S5, TIF file, 0.1 MB. Copyright © 2022 Ma et al. 2022 Ma et al. https://creativecommons.org/licenses/by/4.0/ This content is distributed under the terms of the Creative Commons Attribution 4.0 International license.

10.1128/mbio.02013-22.10 TABLE S5 Primers used in this study. Download Table S5, DOCX file, 0.1 MB. Copyright © 2022 Ma et al. 2022 Ma et al. https://creativecommons.org/licenses/by/4.0/ This content is distributed under the terms of the Creative Commons Attribution 4.0 International license.

The numbers of carious lesions and their severity were evaluated using the Keyes’ score, including enamel only (E), slight dentinal (Ds), moderate dentinal (Dm), and extensive dentinal (Dx) (27). The results showed impaired cariogenicity in the strain overexpressing *actA* compared with the UA159/pDL278 strain, with no significant differences in cariogenicity between the UA159 and UA159 Δ*actA* strains (Table 1). Compared to the group infected by the control UA159/pDL278 strain, infection with the strain overexpressing *actA* was shown to significantly decrease total caries and lesion severity (Fig. 5B and C). No significant differences were observed between the groups infected by the UA159 and UA159 Δ*actA* strains in terms of total caries or caries severity (Fig. 5B and C). In addition, there were more carious lesions on the sulcal surface than on the buccal surface in each of the four groups (Fig. 5C). Concerning caries severity, no Dx carious lesions were observed in the UA159/pDL278*-actA* group, although they were observed in the other three groups. Taken together, these data indicate that ActA impaired the cariogenicity of S. mutans.

**TABLE 1:** Effect of *actA* on the cariogenicity of S. mutans (Keyes’ score)

Infecting strain	Buccal				Sulcal				Proximal			
	E	Ds	Dm	DX	E	Ds	Dm	DX	E	Ds	Dm	DX
*S. mutans* UA159	18.7 ± 3.5	8.8 ± 3.3	6.2 ± 2.1	3.3 ± 1.2	19.7 ± 2.9	15.3 ± 2.7	8.8 ± 2.6	3.5 ± 1.4	5.0 ± 0.9	3.3 ± 0.5	2.2 ± 0.8	0.0
UA159/pDL278	18.5 ± 3.4	10.2 ± 3.1	6.8 ± 2.3	3.8 ± 1.5	19.2 ± 2.3	11.5 ± 2.2	8.8 ± 1.9	3.3 ± 1.0	5.3 ± 1.2	3.5 ± 1.0	2.0 ± 0.9	0.0
UA159/pDL278-*actA*	7.8 ± 2.2^*a*^	3.2 ± 1.5^*a*^	1.0 ± 0.9^*b*^	0.0^*c*^	8.8 ± 1.9^*a*^	3.8 ± 1.2^*a*^	2.0 ± 1.1^*a*^	0.0^*c*^	2.0 ± 1.3^*a*^	0.7 ± 0.5^*a*^	0.0^*a*^	0.0
UA159 Δ*actA*	17.8 ± 2.1	9.5 ± 1.9	6.8 ± 1.5	2.8 ± 1.5	18.0 ± 2.8	10.8 ± 1.5	7.8 ± 1.5	4.2 ± 2.1	4.8 ± 1.6	3.0 ± 0.9	1.8 ± 1.2	0.0

Results are presented as mean ± SD with *n* = 6.

a *P* < 0.0001.

b *P* < 0.01.

c *P* < 0.05.

E, enamel only; Ds, slight dentinal (1/4 of the dentin between the enamel and pulp chamber affected); Dm, moderate dentinal (between 1/4 and 3/4 of the dentin affected); Dx, extensive dentinal (beyond 3/4 of the dentin affected).

## DISCUSSION

LDH is a crucial glycolytic enzyme that reversibly catalyzes the conversion of pyruvate to lactate, using NAD^+^ as a cofactor. Therefore, the abnormal expression and activity of LDH are closely correlated with many diseases. The most extensive study of LDH is in tumors, where its expression and activity are commonly upregulated (28–31). As an important PTM, lysine acetylation regulates LDH activity during tumorigenesis. In human pancreatic cancers, the decreased levels of LDH acetylation result in the activation of LDH enzymatic activity, eventually promoting cancer growth and migration (32). Furthermore, S. mutans increases the magnitude of the drop in pH, the odds of tooth demineralization, and the occurrence of dental caries by the LDH fermentation of dietary carbohydrates. Recently, we found several lysine residues of LDH that are acetylated in the acetylome of S. mutans (Table S1). However, the mechanism and impact of lysine acetylation in LDH have not yet been addressed. To identify the potential KAT modifying LDH, we constructed 15 S. mutans strains overexpressing the GNAT family members and detected the differences in acidogenicity between these GNAT overexpressing strains. The present study showed that the KAT ActA directly acetylates LDH. Additionally, the acetylation of LDH negatively regulates its enzymatic activity, and a subsequent rat caries model showed that *actA* overexpression impaired the cariogenicity of S. mutans.

The application of antibodies with high specificity to the acetylated peptides significantly improved their ability to enrich and identified more acetylated lysine residues in protein acetylome analyses (21, 33). Hundreds of acetylated proteins were identified in different bacterial species with the high sensitivity of mass spectrometers. In the protein acetylome of S. mutans, we found that LDH lysine residues could be acetylated (Table S1). Therefore, we investigated the regulatory mechanism of LDH acetylation. In prokaryotes, protein acetylation is mainly carried out by GNAT family members, such as acetyl-CoA synthetase (Acs) being acetylated by Pat in Salmonella enterica, RNase R being acetylated by YfiQ, RcsB being acetylated by YfiQ in E. coli, GlnR being acetylated by AcuA in Saccharopolyspora erythraea, and others (34–38). The present study showed that ActA, one of the 15 GNAT family members, enzymatically acetylates LDH via an enzymatic mechanism. In addition, 10 lysine acetylation sites were identified by MS (Fig. 3 and 4; Table S3). Notably, lysine acetylation also occurs nonenzymatically. In these cases, Ac-CoA or AcP directly serves as the acetyl donor. A previous study showed that Ac-CoA results in substantial mitochondrial protein lysine acetylation (39). In addition, the purified recombinant Bacillus subtilis acetyl-CoA synthetase (*Bs*AcsA) can be acetylated by Ac-CoA nonenzymatically (40). We used 0.5 mM as the Ac-CoA concentration for the *in vitro* acetylation assays. The results showed that Ac-CoA could not directly acetylate LDH in the absence of ActA (0.5 mM) and could only acetylate LDH in the presence of ActA (Fig. 4). These findings demonstrated that ActA catalyzes the acetylation of LDH.

Lysine acetylation can be used by bacteria to regulate their biological processes for rapidly adapting to environmental changes. As lysine acetylation neutralizes the positive charge on the lysine residues of proteins, this modification can affect their functions by altering protein structure, protein-protein or DNA-protein interactions, and cellular localization (41). Thus far, the lysine acetylation of bacterial enzymes has been shown to exert inhibitory effects on their catalytic activity (42). For example, the lysine acetylation of isocitrate dehydrogenase (ICDH), one of the tricarboxylic acid cycle enzymes, decreases its activity, which may affect energy production and carbon flux regulation in E. coli (43). Acetylated MbtA, an adenylating enzyme in Mycobacterium tuberculosis (*Mtb*), inhibited its enzymatic activity, thereby impairing the ability of *Mtb* to adapt to limited iron conditions (44). Similar inhibitory effects of lysine acetylation on enzymatic activity were observed in other enzymes, including the NhoA, an N-hydroxyarylamine O-acetyltransferase, and the MAT, an adenosylmethionine synthase, in E. coli as well as HcsA, a hexanoyl-CoA synthetase A, and PimA, a pimeloyl-CoA synthetase, in Rhodopseudomonas palustris (45–47). We predicted that the acetylation of LDH would inhibit its activity and would increase the pH of the media, since lactate would not be able to be produced. We screened 15 potential GNATs and identified that ActA was responsible for LDH acetylation (Fig. S1). In a follow-up experiment, we demonstrated that ActA acetylates LDH and inhibits its enzymatic activity, which is responsible for reducing lactic acid production (Fig. 1 and 2). It is worth noting that the UA159 Δ*actA* strains also showed a strong signal of LDH acetylation, similar with that observed in the S. mutans UA159 and UA159/pDL278 strains (Fig. 3A). There is functional redundancy among KATs in S. mutans, one of which may have expanded functions, or there may be additional, unknown KATs that were not studied here and are involved in the acetylation of LDH in the absence of ActA. The protein acetylation is regulated by not only an enzymatic mechanism but also a nonenzymatic mechanism, such as the increased AcP levels due to the activated acetate kinase (Ack)-phosphate acetyltransferase (Pta) pathway in the absence of ActA (48). These regulatory mechanisms may explain the similar signal of LDH acetylation in the S. mutans UA159, UA159/pDL278, and UA159 Δ*actA* strains. Furthermore, the similar LDH acetylation degrees explain the absence of significant differences of phenotypic results, including acidogenicity, aciduricity, and LDH activity, between the S. mutans UA159 and UA159 Δ*actA* strains (Fig. 1 and 3). In addition, the mRNA level of *ldh* significantly increased in overexpression strain UA159/pDL278-*actA*, which may be caused by the impaired LDH activity (Fig. 3B and C). Consistently, there were no significant differences of the *ldh* expression or the LDH activity in UA159 Δ*actA* compared with those of S. mutans UA159. Therefore, there may be a positive feedback loop with some unidentified transcription factor sensing LDH activity and upregulating *ldh* transcription when LDH activity is low.

In the presence of dietary carbohydrates, fermentation products change from mixed acid to lactic acid (49). Thus, the continued metabolism and fermentation of carbohydrates via LDH causes the accumulation of lactic acid within the cytoplasm, acidifying the microenvironment; however, S. mutans has evolved an acid-induced adaption, the acid tolerance response (ATR), to maintain a neutral pH in the cytoplasm (50, 51). F-type ATPase (the alpha-subunit of the proton translocator is encoded by *atpD*) proton pumps are central to the ability of S. mutans to maintain pH homeostasis (52, 53). The present study showed that the overexpression of ActA decreases lactic acid production of S. mutans, which might be attributable to the impaired aciduricity (Fig. 2). For example, at low pH, S. mutans increases the activity of F-type ATPase and the subsequent expulsion of protons from the cell, which helps maintain an elevated intracellular pH, relative to its surroundings (54). The antimicrobial peptide GH12 inhibits the acidogenicity of S. mutans and correspondingly impairs its aciduricity by inhibiting the enzymatic activity of F-type ATPase (55). The activity of F-type ATPase is paramount to aciduricity in various species, such that this enzyme is directly linked to the bacterial ability to survive in acidic conditions (6, 56–58). This involves the hemostasis between acidogenicity and aciduricity mechanisms, which prevents S. mutans from becoming a victim of its own metabolism. However, the mechanisms of the coordinated linkage between acidogenicity and aciduricity have not yet been fully elucidated, and further investigation is necessary.

The in-depth acetylome profiles of S. mutans demonstrated that the lysine residues of LDH are acetylated *in vivo* (Table S1). In this study, we identified and characterized that ActA, a GNAT family member, with KAT activity could acetylate LDH and that ActA-dependent acetylation of LDH negatively impacted its enzymatic activity, which subsequently reduced the production of lactic acid in S. mutans. In addition, 10 lysine acetylation sites have been identified *in vitro* by MS, the majority (70%) of which have also been detected *in vivo*. How these identified lysine acetylation sites impact its activity is an interesting question, and ongoing studies in our laboratory are focused on answering this question. Our findings provide a starting point for the further analysis of the functions and regulatory mechanisms of lysine acetylation modification in S. mutans metabolism and virulence.

## MATERIALS AND METHODS

### Bacterial strains and growth conditions.

S. mutans UA159 was commercially obtained from the American Type Culture Collection (ATCC, Manassas, VA, USA) and cultured in brain heart infusion (BHI) broth (Difco, Sparks, MD, USA) under anaerobic conditions (85% N_2_, 5% CO_2_, and 10% H_2_) at 37°C. For the biofilm assay, 1% sucrose (Sigma, 1%, wt/vol) was added to the BHI medium (BHIS). Competent Escherichia coli BL21(DE3) and DH5α cells were purchased from Tsingke Corporation (Beijing, China) and were routinely grown in Luria-Bertani LB medium (BD, Sparks, MD, USA) aerobically (95% air, 5% CO_2_). In addition, antibiotics were added to growth media at the following concentrations: kanamycin (1.0 mg/mL for S. mutans and 30 μg/mL for E. coli), erythromycin (10 μg/mL for S. mutans and 300 μg/mL for E. coli), and spectinomycin (1.0 mg/mL for S. mutans and 300 μg/mL for E. coli), when necessary. Table S4 lists all of the strains and plasmids used in this study.

10.1128/mbio.02013-22.9 TABLE S4 Bacterial strains and plasmids used in this study. Download Table S4, DOCX file, 0.1 MB. Copyright © 2022 Ma et al. 2022 Ma et al. https://creativecommons.org/licenses/by/4.0/ This content is distributed under the terms of the Creative Commons Attribution 4.0 International license.

### Construction of overexpression strains.

The genes of the GNAT family members (*actA*-*actO*) listed in Table S4 were amplified from S. mutans genomic DNA via PCR. All of the primers were designed using an online tool provided by TaKaRa (https://www.takarabio.com/learning-centers/cloning/primer-design-and-other-tools) and are listed in Table S5. First, the PCR products were purified and cloned into the linearized E. coli-Streptococcus shuttle vector pDL278 at the same insertion site (sequence 6,360 to 6,381) using an In-Fusion HD cloning kit (TaKaRa, Japan). Then, the resultant plasmids were transformed into S. mutans UA159 to generate the overexpression strains (UA159/pDL278-*actA* to UA159/pDL278-*actO*) of the GNAT family members. Relative overexpressing strains were selected using plates containing spectinomycin (1 mg/mL) and were verified via PCR and sequencing.

### Construction of markerless in-frame deletion mutants.

The markerless in-frame deletion mutant of the S. mutans GNAT family member ActA was constructed using a previously described two-step transformation method (59, 60). Table S5 lists all of the primers designed in this study. First, approximately 1 kb upstream and downstream of the *actA* open reading frame, was amplified from S. mutans UA159 genomic DNA using PCR with the upF/upR and dnF/dnR primers. Next, the selection cassette IFDC2 (positive for erythromycin and negative for *p*-Cl-Che) was amplified by the ldhF/ermR primers. The fragments containing the overlapping regions were then ligated using overlap extension PCR with the upF/dnR primers and transformed into S. mutans UA159. The markerless in-frame deletion mutant of *actA* was selected using plates containing erythromycin (12 μg/mL). Concerning the second transformation, upstream and downstream fragments of *actA* were amplified using PCR with the upF/updnR and dnF/dnR primers and ligated using overlap extension PCR. The ligated fragment was transformed into the mutant strain containing IFDC2 and selected using plates containing *p*-Cl-Che (4 mg/mL). Finally, the markerless in-frame deletion mutant was confirmed via PCR and sequencing.

### Glycolytic pH drop and lactic acid production experiments.

The ability of S. mutans to lower the pH through glycolysis was monitored as described previously (57), with some modifications. Briefly, S. mutans was harvested at an OD_600 nm_ of 0.5, washed with 1 mL of a phosphate-buffered saline (PBS) solution of 8 mM Na_2_HPO_4_, 2 mM KH_2_PO_4_, 137 mM NaCl, and 2.7 mM KCl (pH 7.4), and resuspended in a salt solution of 50 mM KCl and 1 mM MgCl_2_ (pH 7.2). The decrease in pH was initiated by adding 1% (wt/vol) glucose, which was monitored at 5 min or 10 min intervals for 60 min using a Benchtop meter (Thermo Scientific, Waltham, MA).

S. mutans was diluted with BHIS to a final concentration of 1 × 10^6^ CFU/mL and anaerobically cultured in 24-well plates for 24 h for the lactic acid production experiment. The biofilms were washed with PBS twice and mixed with 1.5 mL buffered peptone water (BPW, KuanKai, Guangzhou, China) supplemented with 0.2% sucrose. The plates were further incubated for 120 min at 37°C anaerobically. After removing planktonic cells by centrifugation, the supernatants were decanted to measure their lactic acid concentrations using a lactic acid assay kit (Solarbio, Beijing, China). The absorbance was recorded at 570 nm using a microplate spectrophotometer, and the lactic acid concentrations were calculated using standard curves.

### Biofilm formation assays.

The biofilm biomass was assessed using the crystal violet (CV) staining method described previously (61). Briefly, after fixing the biofilms with methanol for 15 min and staining with 0.01% (wt/vol) CV for 5 min, the dye bound to the cells was resolubilized with 33% (vol/vol) acetic acid. The absorbance of the solution was measured at 575 nm. Concerning the CFU counts, after resuspending the cells from 24 h biofilms, the cells were serially diluted 10^6^-fold, cultured on BHI agar plates, and anaerobically incubated at 37°C for 48 h before being used to determine CFU counts.

### Acid killing assays.

The strains were pregrown as described above to test their acid resistance ability. When the cells grew to an OD_600nm_ of 0.5, they were harvested and washed once with 0.1 M glycine (pH 7.2) and resuspended in 0.1 M glycine (pH 2.8). The samples were stirred continuously at room temperature, and cell aliquots were harvested at 15, 30, and 45 min. The killing process was terminated with PBS. Then, the cells were serially diluted, cultured on BHI-agar plates, and incubated at 37°C for 48 h before the CFU were counted at each time interval.

### LDH activity assays.

S. mutans were grown to an OD_600nm_ of 0.5, and cell pellets were harvested. The protein concentrations were quantified using a BCA protein assay kit (Beyotime, Shanghai, China), and the LDH activity was detected and calculated using an LDH activity kit (Solarbio, Beijing, China), following the manufacturers’ instructions. The absorbance of LDH was recorded at 450 nm, and the LDH activity in the control group was normalized to 1.0.

### Purification of recombinant ActA and LDH.

*actA and ldh* were amplified from S. mutans genomic DNA by PCR and purified. The products were digested by EcoRI and XhoI and then cloned into the expression vector pET28a (Novagen) with an N-terminal fusion of a 6×His tag. Next, the reconstructed plasmids were transformed into E. coli BL21(DE3) cells. The proteins were purified, and their concentrations were determined as described previously (61). First, overnight cultures of the transformant were diluted 1:20 with fresh LB medium containing 50 μg/mL of kanamycin until an OD_600nm_ of 0.6 was achieved. After further growth with 1 mM isopropyl-β-d-thiogalactopyranoside (IPTG) at 37°C for 6 h to induce protein expression, the cell pellets were harvested and lysed via sonication. Then, the recombinant ActA and LDH were purified using a His-tagged protein purification kit (Beyotime, Shanghai, China) as per the manufacturer’s instructions and were concentrated via 10 kDa MWKO ultrafiltration (Millipore Amincon, Merck, Germany) from the cell debris. The purified ActA and LDH were confirmed by SDS-PAGE and stored at −80°C for future use.

### LC-MS/MS analysis.

The identification of LDH and its lysine acetylation were completed using LC-MS/MS analysis. For in-gel tryptic digestion, gel pieces were destained in 50 mM NH_4_HCO_3_ in 50% acetonitrile (vol/vol) until clear. The gel pieces were dehydrated with 100 μL of 100% acetonitrile for 5 min, the liquid was removed, and the gel pieces were rehydrated in 10 mM dithiothreitol and incubated at 56°C for 60 min. Then, the gel pieces were again dehydrated in 100% acetonitrile, the liquid was removed, and the gel pieces were rehydrated with 55 mM iodoacetamide. The samples were incubated at room temperature in the dark for 45 min. The gel pieces were then washed with 50 mM NH_4_HCO_3_, dehydrated with 100% acetonitrile, and rehydrated with 10 ng/μL trypsin resuspended in 50 mM NH_4_HCO_3_ on ice for 1 h. The excess liquid was removed, and the gel pieces were digested with trypsin at 37°C overnight. Peptides were extracted with 50% acetonitrile/5% formic acid, followed by 100% acetonitrile. The peptides were dried to completion and then resuspended in 2% acetonitrile/0.1% formic acid.

The tryptic peptides were dissolved in 0.1% formic acid (solvent A), directly loaded onto a homemade reverse-phase analytical column (15 cm in length, 75 μm in diameter). The gradient was comprised of an increase from 6% to 23% solvent B (0.1% formic acid in 98% acetonitrile) over 16 min, 23% to 35% for 8 min, and increasing to 80% for 3 min, followed by maintenance at 80% for the last 3 min, all at a constant flow rate of 400 nL/min on an EASY-nLC 1000 UPLC system. Next, the peptides were subjected to a nanospray ionization (NSI) source, followed by tandem mass spectrometry (MS/MS) in a Q Exactive Plus system (Thermo) that was coupled online to the UPLC. The applied electrospray voltage was 2.2 kV. The *m/z* scan range was 350 to 1,800 for a full scan, and intact peptides were detected in the Orbitrap at a resolution of 70,000. The peptides were then selected for MS/MS using the NCE setting at 28, and the fragments were detected in the Orbitrap at a resolution of 17,500. A data-dependent procedure that alternated between one MS scan was followed by 20 MS/MS scans with 15.0 s dynamic exclusion. The automatic gain control (AGC) was set at 5E4.

The Proteome Discoverer 1.3 software package was used to process the collected MS/MS data. Tandem mass spectra were searched against the S. mutans database. Trypsin/P was specified as a cleavage enzyme, allowing up to two missing cleavages. The mass error was set to 10 ppm for precursor ions and 0.02 Da for fragment ions. Carbamidomethyl on Cys was specified as a fixed modification, and oxidation on Met and acetylation on Lys were specified as variable modifications. The peptide confidence was set at high, and the peptide ion score was set at >20.

### Western blotting.

Western blotting was performed as described previously (62). The protein concentrations were determined using the BCA protein assay kit (Beyotime), following the manufacturer’s instructions. Equal amounts of protein (30 μg) were mixed with SDS-PAGE sample buffer, boiled for 10 min, and separated using 8% SDS-PAGE (110 V). The polypeptides were electrophoretically transferred to a polyvinylidene difluoride (PVDF) membrane blocked in 5% (wt/vol) nonfat dry milk at room temperature for 1 h. Then, the membranes were incubated with anti-acetyl lysine (Anti-Ac) antibody and diluted 1:1000 in TBST at 4°C overnight. After six rounds of washing with TBST, the Anti-Ac membranes were incubated with HRP-conjugated goat anti-rabbit secondary antibody at a 1:10000 dilution in TBST at room temperature for 2 h. The membranes were observed with the immobilon Western Chemiluminescent HRP substrate kit (Millipore). Images were captured with the Bio-Rad GS-700 imaging densitometer and quantified using ImageJ software. Polyclonal antibodies to gyrase were used as a control for whole-cell extracts. The upregulated band was analyzed by LC-MS/MS.

### *In vitro* acetylation analysis.

*In vitro* acetylation analysis was performed as described (63), with minor modifications. Briefly, the purified LDH was incubated with Ac-CoA in total reaction volume of 20 μL, containing 100 mM Tris-HCl (pH 7.5), 150 mM NaCl, and 10 mM MgCl_2_ in the presence or absence of ActA. The reactions were allowed to occur at 37°C for 3 h and were terminated by the addition of SDS sample buffer or by boiling in SDS sample buffer. Then, the samples were analyzed by SDS-PAGE, Western blotting, and an LDH activity detection kit (Solarbio). In addition, the acetylated and nonacetylated bands were anlyzed by LC-MS/MS.

### RNA extraction and qRT-PCR.

Total RNA was extracted and purified as described previously (64). First, RNA (1 μg) was reverse-transcribed to cDNA using a cDNA synthesis kit (TaKaRa, Shiga, Japan). Then, quantitative real-time PCR (qRT-PCR) was performed using SYBR green master mix on a Bio-Rad CFX96 system (Bio-Rad). Table S5 lists all of the primers used in this study. The relative expression levels of genes were calculated using the 2^-ΔΔCt^ method with values normalized to the reference gene 16S rRNA.

### Rat caries model.

The rat caries model was performed as previously described (65), with some modifications. Twenty-four 3-week-old, female, specific pathogen-free (SPF) Sprague Dawley (SD) rats (Chengdu Dossy Experimental Animals Co., Ltd., China), each weighing 50 ± 5 g, were purchased. The rats were provided with distilled water with ampicillin (1 g/kg) to drink for the first 3 days and distilled water without ampicillin for another day to elute the antibiotic. Then, the rats were randomly assigned to four groups (*n* = 6), and each group was incubated with the UA159, UA159/pDL278, UA159/pDL278*-acA*, or UA159 Δ*actA* strain by oral swabbing with a fresh overnight culture for three consecutive days. Meanwhile, all of the rats in each group were provided with the cariogenic diet 2000 (*TrophicDiet*, Trophic Animal Feed, Suzhou, China) and sterile water containing 5% (wt/vol) sucrose *ad libitum*. Oral swab samples were collected 5 days after contamination to confirm colonization. All of the rats were weighed every 3 days, and their behavior, capacity to drink/eat, and physical appearance were checked daily until the termination of the experiment. 3 weeks after incubation, all of the rats were sacrificed by carbon dioxide euthanasia, and their maxillae and mandibles were obtained. Plaque samples were collected via sonication in sterile PBS and divided into two parts, one part for verifying the strains of UA159/pDL278 and UA159/pDL278-*actA* by PCR and the other part plated on mitis salivarius agar (Difco) plus bacitracin (Sigma) for counting the bacterial loads. After removing the flesh from the jaws, all of the molar teeth were stained with 0.4% ammonium purpurate for 6 h and then hemi-sectioned with a cutter. Images were captured under a stereomicroscope (Leica EZ4HD; Leica Microsystems AG, Heerbrugg, Switzerland) and scored for carious lesions by the Keyes’ method (27).

### Ethics statement.

The rat experiments in this study were performed in strict accordance with the protocols and procedures approved by the Institutional Animal Care and Use Committee of West China Hospital of Stomatology, Sichuan University (WCHSIRB-D-2021-156). The animal care and protocol adhered to the Chinese National Laboratory Animal-Guidelines for Ethical Review of Animal Welfare.

### Statistical analysis.

All of the experiments were performed in triplicate and were reproduced at least three times. The data were analyzed using SPSS 20.0 (SPSS Software Inc, Chicago, IL, USA) and Prism 9.0 (GraphPad Software Inc, San Diego, CA, USA). The Student's *t* test was used for two-by-two comparisons of the groups. A one-way analysis of variance (ANOVA) and Tukey’s test were performed to compare data between multiple groups. A value of *P* < 0.05 was considered to be indicative of a statistically significant result.
