# Supplementary material for: Acetylation of Lactate Dehydrogenase Negatively Regulates the Acidogenicity of Streptococcus mutans
Source: mBio. 2022 Aug 31;13(5):e02013-22. doi: 10.1128/mbio.02013-22 (PMC9600946; doi:10.1128/mbio.02013-22)
Supplement: TABLE S1 [file mbio.02013-22-s0006.docx]

**TABLE S1** Identified lysine acetylation sites of LDH in the acetylome profiles of *S. mutans*.

| **Accession** | **Protein names** | **MW [kDa]** | **Position** | **Peptide score** | **Modified sequence** | **Reference** |
| --- | --- | --- | --- | --- | --- | --- |
| P26283 | P26283 | 35.22329578 | K 319 | 141.13 | _AIIDEAFSk(Ac)*EEFAAAAR_ | (22) |
| P26283 | P26283 | 35.22329578 | K 64 | 101.5 | _AVGDALDLSHALAFTSPKk(Ac)*K_ |  |
| P26283 | P26283 | 35.22329578 | K 231 | 99.752 | _DAAYTIINk(Ac)*K_ |  |
| P26283 | P26283 | 35.22329578 | K 310 | 75.764 | _ELk(Ac)*AIIDEAFSK_ |  |
| P26283 | P26283 | 35.22329578 | K 100 | 147.26 | _LDLVGk(Ac)*NLAINK_ |  |
| P26283 | P26283 | 35.22329578 | K 307 | 57.164 | _MQASAk(Ac)*ELK_ |  |
| P26283 | P26283 | 35.22329578 | K 299 | 127.36 | _PVNIPLNDAEk(Ac)*QK_ |  |
| P26283 | P26283 | 35.22329578 | K 165 | 136.83 | _QALAEKk(Ac)*LDVDAR_ |  |

* Indicating lysine acetylation sites of LDH identified in the acetylome profiles of *S. mutans*.
