# Supplementary figures and images for: Acetylation of Lactate Dehydrogenase Negatively Regulates the Acidogenicity of Streptococcus mutans
Source: mBio. 2022 Aug 31;13(5):e02013-22. doi: 10.1128/mbio.02013-22 (PMC9600946; doi:10.1128/mbio.02013-22)

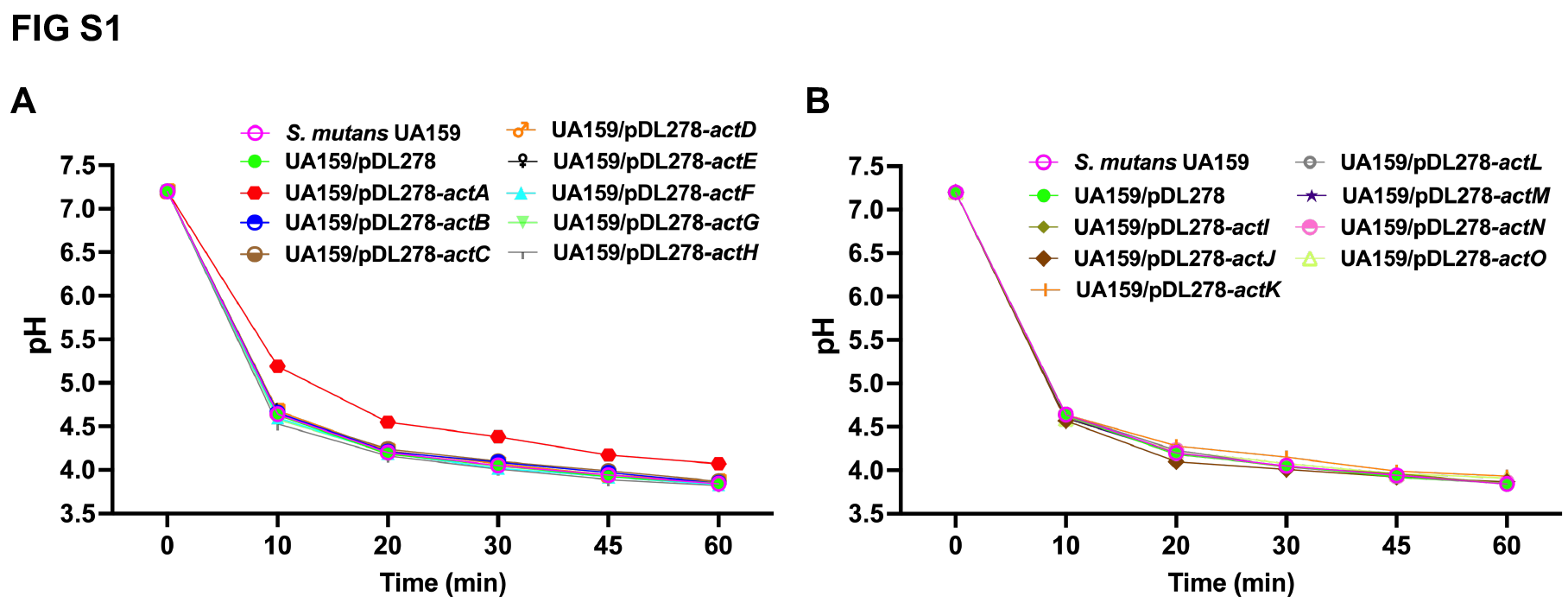

Supplement: FIG S1 [file mbio.02013-22-s0001.tif]

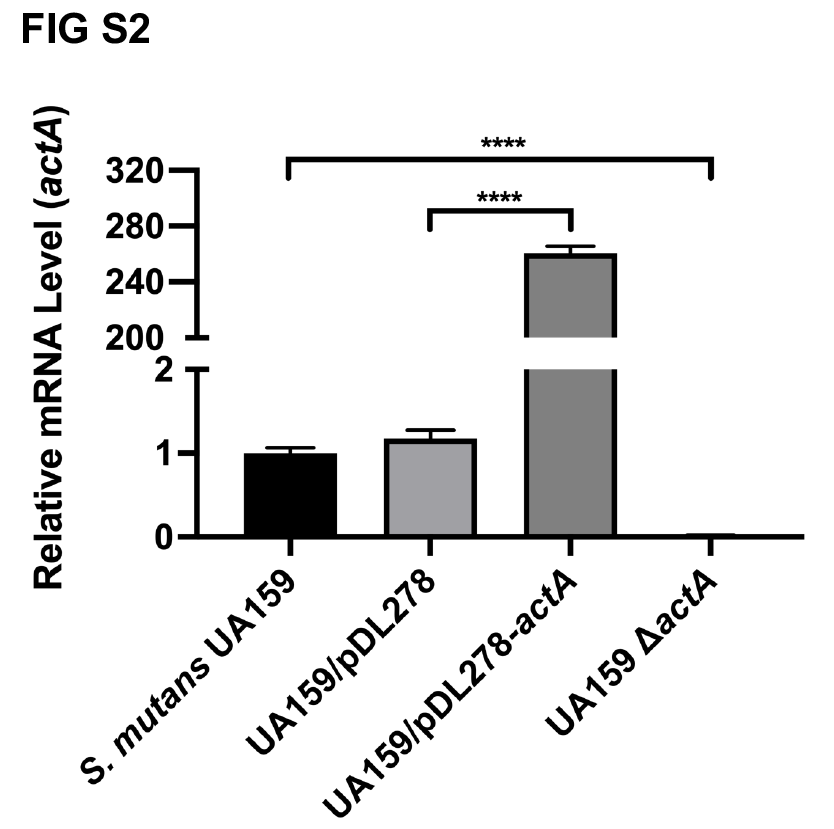

Supplement: FIG S2 [file mbio.02013-22-s0002.tif]

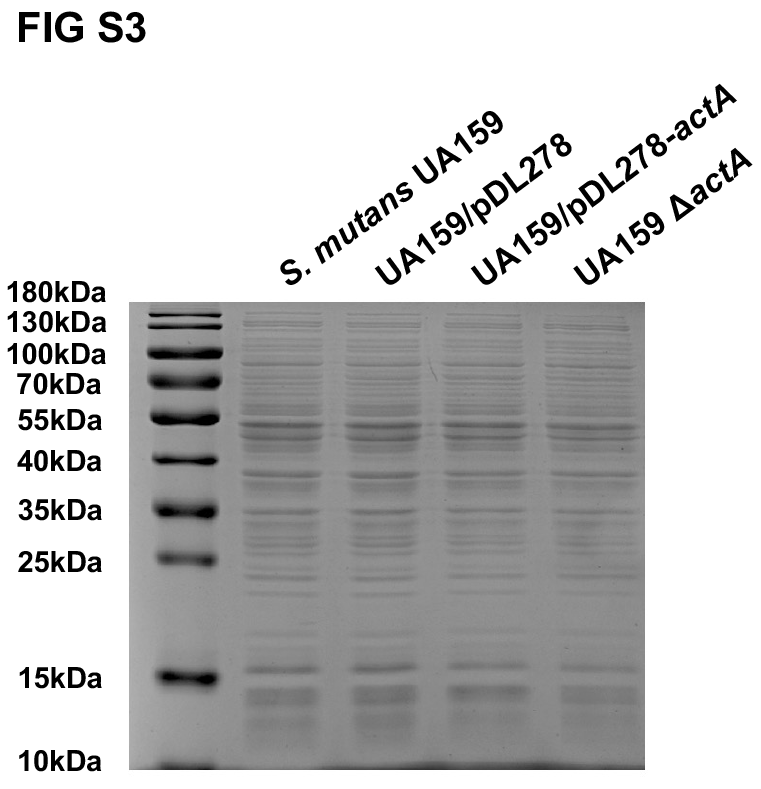

Supplement: FIG S3 [file mbio.02013-22-s0003.tif]

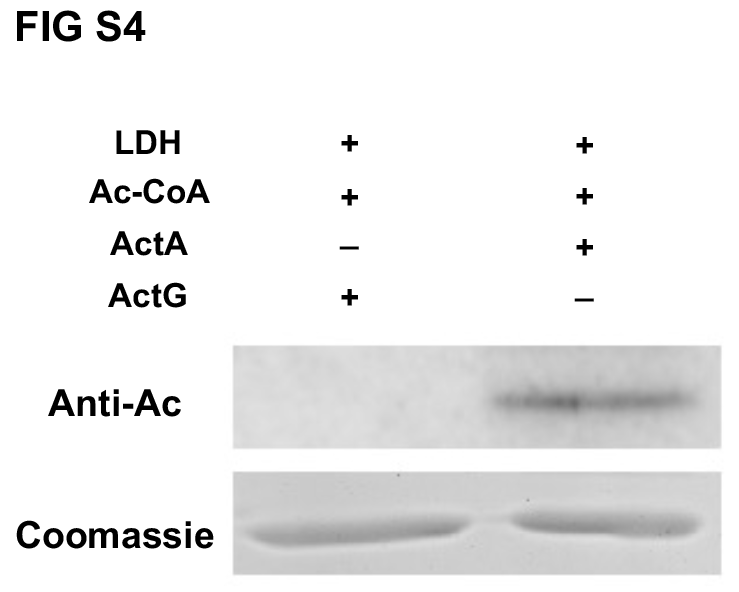

Supplement: FIG S4 [file mbio.02013-22-s0004.tif]

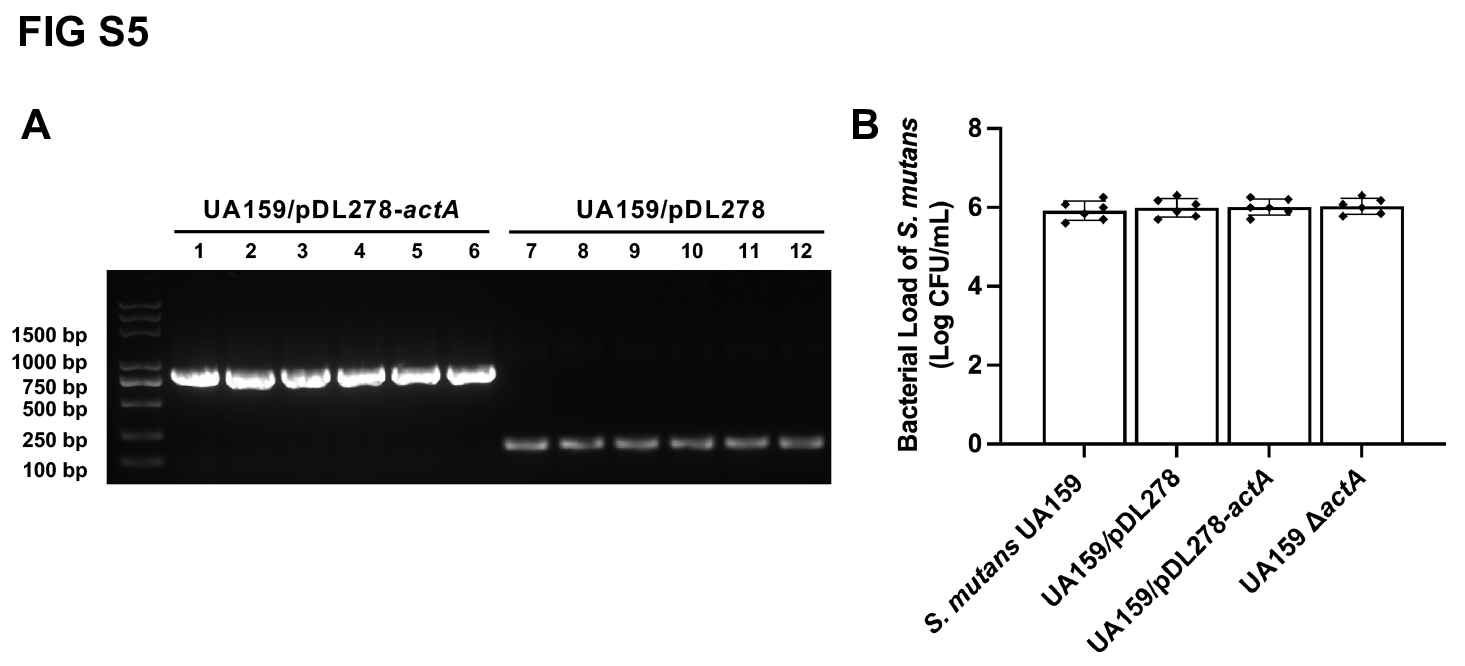

Supplement: FIG S5 [file mbio.02013-22-s0005.tif]
