# Supplementary material for: Acetylation of Lactate Dehydrogenase Negatively Regulates the Acidogenicity of Streptococcus mutans
Source: mBio. 2022 Aug 31;13(5):e02013-22. doi: 10.1128/mbio.02013-22 (PMC9600946; doi:10.1128/mbio.02013-22)
Supplement: TABLE S2 [file mbio.02013-22-s0007.docx]

**TABLE S2** Identified target substrate (LDH) of ActA by MS analysis.

| **Accession** | **Protein names** | **Gene names** | **MW [kDa]** | **Protein score** | **Sequence coverage (%)** | **Unique Peptides** | **Peptides** | **PSMs** |
| --- | --- | --- | --- | --- | --- | --- | --- | --- |
| P26283 | L-lactate dehydrogenase | ldh | 35.22 | 3758.99 | 79.53 | 25 | 25 | 75 |
| P72483 | Elongation factor Tu | tuf | 43.89 | 771.84 | 45.48 | 16 | 16 | 22 |
| Q8DTS9 | Enolase | eno | 46.83 | 739.12 | 30.09 | 11 | 11 | 14 |
| Q8DTG0 | Putative oxidoreductase | SMU_1387 | 36.76 | 552.89 | 40.8 | 12 | 12 | 13 |
| Q8DVV4 | Elongation factor G | fusA | 76.62 | 552.15 | 26.55 | 12 | 12 | 13 |
| Q8DVD4 | Putative cell division protein DivIVA | divIVA | 30.8 | 514.79 | 46.86 | 10 | 10 | 11 |
| Q8DW43 | Ketol-acid reductoisomerase (NADP(+)) | ilvC | 37.26 | 482.08 | 39.12 | 12 | 12 | 13 |
| P95780 | dTDP-glucose 4,6-dehydratase | rmlB | 39.26 | 474.93 | 35.34 | 11 | 11 | 14 |
| Q8DT50 | DAGKc domain-containing protein | SMU_1542c | 38.29 | 472.76 | 28.82 | 9 | 9 | 10 |
| Q8DSE4 | Putative aminopeptidase P | pepP | 39.67 | 459.14 | 31.07 | 11 | 11 | 11 |
| Q8DUW0 | Putative prephenate dehydrogenase | SMU_781 | 41.56 | 455.06 | 33.97 | 11 | 11 | 12 |
| Q8DUW2 | 3-dehydroquinate synthase | aroB | 39.04 | 446.07 | 33.52 | 11 | 11 | 12 |
