# Supplementary material for: Acetylation of Lactate Dehydrogenase Negatively Regulates the Acidogenicity of Streptococcus mutans
Source: mBio. 2022 Aug 31;13(5):e02013-22. doi: 10.1128/mbio.02013-22 (PMC9600946; doi:10.1128/mbio.02013-22)
Supplement: TABLE S3 [file mbio.02013-22-s0008.docx]

**TABLE S3** Identified lysine acetylation sites of LDH by MS/MS fragmentation *in vitro* acetylation analysis.

| **Accession** | **Protein names** | **MW [kDa]** | **Protein score** | **Sequence coverage (%)** | **Position** | **Peptide score** | **Modified sequence** |
| --- | --- | --- | --- | --- | --- | --- | --- |
| P26283 | P26283 | 35.22329578 | 4806.48 | 47.56 | K 165^a^ | 93.18 | QALAEk(Ac)*LDVDAR |
| P26283 | P26283 | 35.22329578 | 4806.48 | 47.56 | K 319^a^ | 88.93 | AIIDEAFSk(Ac)*EEFAAAAR |
| P26283 | P26283 | 35.22329578 | 4806.48 | 47.56 | K 299^a^ | 76.29 | PVNIPLNDAEk(Ac)*QK |
| P26283 | P26283 | 35.22329578 | 4806.48 | 47.56 | K 145 | 75.7 | FSGFPAEk(Ac)*VIGSGTSLDTAR |
| P26283 | P26283 | 35.22329578 | 4806.48 | 47.56 | K 100^a^ | 53.55 | LDLVGk(Ac)*NLAINK |
| P26283 | P26283 | 35.22329578 | 4806.48 | 47.56 | K 307^a^ | 51.91 | MQASAk(Ac)*ELK |
| P26283 | P26283 | 35.22329578 | 4806.48 | 47.56 | K 310^a^ | 50.09 | ELk(Ac)*AIIDEAFSK |
| P26283 | P26283 | 35.22329578 | 4806.48 | 47.56 | K 231^a^ | 39.75 | DAAYTIINk(Ac)*K |
| P26283 | P26283 | 35.22329578 | 4806.48 | 47.56 | K 65 | 38.75 | k(Ac)*IYAAK |
| P26283 | P26283 | 35.22329578 | 4806.48 | 47.56 | K 301 | 37.56 | Qk(Ac)*MQASAK |

* Indicating lysine acetylation sites of LDH identified *in vitro* acetylation analysis.

^a^ Indicating lysine acetylation sites of LDH also identified *in vivo*.
