# Supplementary material for: Acetylation of Lactate Dehydrogenase Negatively Regulates the Acidogenicity of Streptococcus mutans
Source: mBio. 2022 Aug 31;13(5):e02013-22. doi: 10.1128/mbio.02013-22 (PMC9600946; doi:10.1128/mbio.02013-22)
Supplement: TABLE S5 [file mbio.02013-22-s0010.docx]

**TABLE S5** Primers used in this study.

| **Primers** | **Sequence (5’ to 3’)** | **Used in** |
| --- | --- | --- |
| pDL278 F | GTCGACCTGCAGGCATGC | Overexpression |
| pDL278 R | GAGCTCGAATTCACTGGCCG | Overexpression |
| *actA* LDHF | CGGCCAGTGAATTCGAGCTCAGAGCCCGAGCAACAATAACACT | Overexpression |
| *actA* LDHR | CTTTCATCATGTTCTAAACATCTCCTTATAATTT | Overexpression |
| *actA* overF | TGTTTAGAACATGATGAAAGATGAGAAAAACCAAG | Overexpression |
| *actA* overR | TTGCATGCCTGCAGGTCGACTTAATAATCTATTAAACCTTCACGT | Overexpression |
| *actB* LDHF | CGGCCAGTGAATTCGAGCTCAGAGCCCGAGCAACAATAACACT | Overexpression |
| *actB* LDHR | TGGTAGTCATGTTCTAAACATCTCCTTATAATTT | Overexpression |
| *actB* overF | TGTTTAGAACATGACTACCACTATCCGTCA | Overexpression |
| *actB* overR | TTGCATGCCTGCAGGTCGACTTAGGAAAAATAATCTATTCCCTCT | Overexpression |
| *actC* LDHF | CGGCCAGTGAATTCGAGCTCAGAGCCCGAGCAACAATAACACT | Overexpression |
| *actC* LDHR | TTAAAGTCATGTTCTAAACATCTCCTTATAATTT | Overexpression |
| *actC* overF | TGTTTAGAACATGACTTTAACTCAACAAAAAGTG | Overexpression |
| *actC* overR | TTGCATGCCTGCAGGTCGACCTATACTGTTATTAGCACTAAGC | Overexpression |
| *actD* LDHF | CGGCCAGTGAATTCGAGCTCAGAGCCCGAGCAACAATAACACT | Overexpression |
| *actD* LDHR | TAACTGGCATGTTCTAAACATCTCCTTATAATTT | Overexpression |
| *actD* overF | TGTTTAGAACATGCCAGTTAATCAGTATCAG | Overexpression |
| *actD* overR | TTGCATGCCTGCAGGTCGACTTAACAATCTTTTAATGCTTTTATC | Overexpression |
| *actE* LDHF | CGGCCAGTGAATTCGAGCTCAGAGCCCGAGCAACAATAACACT | Overexpression |
| *actE* LDHR | TTATTTTCATGTTCTAAACATCTCCTTATAATTT | Overexpression |
| *actE* overF | TGTTTAGAACATGAAAATAAGAAATGCTTGTAAA | Overexpression |
| *actE* overR | TTGCATGCCTGCAGGTCGACTTAATCAAGCAAACGTTTTTG | Overexpression |
| *actF* LDHF | CGGCCAGTGAATTCGAGCTCAGAGCCCGAGCAACAATAACACT | Overexpression |
| *actF* LDHR | TCACTGCCATGTTCTAAACATCTCCTTATAATTT | Overexpression |
| *actF* overF | TGTTTAGAACATGGCAGTGAAAGAACCGC | Overexpression |
| *actF* overR | TTGCATGCCTGCAGGTCGACTTAAAACCAGCGCCTTTGC | Overexpression |
| *actG* LDHF | CGGCCAGTGAATTCGAGCTCAGAGCCCGAGCAACAATAACACT | Overexpression |
| *actG* LDHR | GAACGATCATGTTCTAAACATCTCCTTATAATTT | Overexpression |
| *actG* overF | TGTTTAGAACATGATCGTTCATTTTTCAG | Overexpression |
| *actG* overR | TTGCATGCCTGCAGGTCGACTTATAACTTATAGACAAAATAATAA | Overexpression |
| *actH* LDHF | CGGCCAGTGAATTCGAGCTCAGAGCCCGAGCAACAATAACACT | Overexpression |
| *actH* LDHR | TAATAGACATGTTCTAAACATCTCCTTATAATTT | Overexpression |
| *actH* overF | TGTTTAGAACATGTCTATTAGATTGGCACA | Overexpression |
| *actH* overR | TTGCATGCCTGCAGGTCGACTTAAAGAATTTTCTCCATTTTCGT | Overexpression |
| *actI* LDHF | CGGCCAGTGAATTCGAGCTCAGAGCCCGAGCAACAATAACACT | Overexpression |
| *actI* LDHR | CGCCGTACATGTTCTAAACATCTCCTTATAATTT | Overexpression |
| *actI* overF | TGTTTAGAACATGTACGGCGGAGCTGGT | Overexpression |
| *actI* overR | TTGCATGCCTGCAGGTCGACTCATATGCTGTTCCTTTCAGTCACA | Overexpression |
| *actJ* LDHF | CGGCCAGTGAATTCGAGCTCAGAGCCCGAGCAACAATAACACT | Overexpression |
| *actJ* LDHR | CCTGTGACATGTTCTAAACATCTCCTTATAATTT | Overexpression |
| *actJ* overF | TGTTTAGAACATGTCACAGGTTGAAATTAGAAAAG | Overexpression |
| *actJ* overR | TTGCATGCCTGCAGGTCGACCTAACTTAAAGACTTTTTAAGCAAC | Overexpression |
| *actK* LDHF | CGGCCAGTGAATTCGAGCTCAGAGCCCGAGCAACAATAACACT | Overexpression |
| *actK* LDHR | ATATATCCATGTTCTAAACATCTCCTTATAATTT | Overexpression |
| *actK* overF | TGTTTAGAACATGGATATATGGACACAGTTAGGAC | Overexpression |
| *actK* overR | TTGCATGCCTGCAGGTCGACTTACTCATAACTTTTATCACCTGCT | Overexpression |
| *actL* LDHF | CGGCCAGTGAATTCGAGCTCAGAGCCCGAGCAACAATAACACT | Overexpression |
| *actL* LDHR | GGACTGCCATGTTCTAAACATCTCCTTATAATTT | Overexpression |
| *actL* overF | TGTTTAGAACATGGCAGTCCTCAGTTCTG | Overexpression |
| *actL* overR | TTGCATGCCTGCAGGTCGACTTAAAAGCCATGATAATCTGCTTCA | Overexpression |
| *actM* LDHF | CGGCCAGTGAATTCGAGCTCAGAGCCCGAGCAACAATAACACT | Overexpression |
| *actM* LDHR | GAATAAGCATGTTCTAAACATCTCCTTATAATTT | Overexpression |
| *actM* overF | TGTTTAGAACATGCTTATTCGACAAGTGACAATG | Overexpression |
| *actM* overR | TTGCATGCCTGCAGGTCGACCTAAATCTTAGGATTTTCCCAAATC | Overexpression |
| *actN* LDHF | CGGCCAGTGAATTCGAGCTCAGAGCCCGAGCAACAATAACACT | Overexpression |
| *actN* LDHR | TTATTTTCATGTTCTAAACATCTCCTTATAATTT | Overexpression |
| *actN* overF | TGTTTAGAACATGAAAATAAGCCCTATGTTAC | Overexpression |
| *actN* overR | TTGCATGCCTGCAGGTCGACTTATTTGGCATAGGCAGC | Overexpression |
| *actO* LDHF | CGGCCAGTGAATTCGAGCTCAGAGCCCGAGCAACAATAACACT | Overexpression |
| *actO* LDHR | ATTTTAACATGTTCTAAACATCTCCTTATAATTT | Overexpression |
| *actO* overF | TGTTTAGAACATGTTAAAATTACGTAGACCGACT | Overexpression |
| *actO* overR | TTGCATGCCTGCAGGTCGACTCACTCTTCCACTCTCCTGGT | Overexpression |
| *actA* upF | CCTAGCAGATTGGTACAG | In-frame deletion construction |
| *actA* upR | GAGTGTTATTGTTGCTCGGCATTACACCCGTTTGACA | In-frame deletion construction |
| *actA* dnF | GGTATACTACTGACAGCTTCGATAAGAAAGACATATGATAG | In-frame deletion construction |
| *actA* dnR | CATATTCAACGGCCGCAG | In-frame deletion construction |
| *actA* checkF | AATCTACTCATCTGTGAAGAG | In-frame deletion construction |
| *actA* checkR | TAGCATTTAGATCGAGTTCTG | In-frame deletion construction |
| *actA* updnR | CTATCATATGTCTTTCTTATCCATTACACCCGTTTGACA | In-frame deletion construction |
| ActA protein F | AAATTTGAATTCATATGATGAAAGATGAGAAAAACCA | Protein expression |
| ActA protein R | ATATATCTCGAGTTAATAATCTATTAAACCTTCAC | Protein expression |
| LDH protein F | AGGAGGGAATTCATATGACTGCAACTAAACAAC | Protein expression |
| LDH protein R | TATATACTCGAGTTAGTTACGAGCTGCAGCAGCAAATTC | Protein expression |
| *Ldh* F | CCGAGCAACAATAACACTC | In-frame deletion construction |
| *Erm* R | GAAGCTGTCAGTAGTATACC | In-frame deletion construction |
| *16S* F | CCATGTGTAGCGGTGAAATGC | qRT-PCR |
| *16S* R | TCATCGTTTACGGCGTGGAC | qRT-PCR |
| *ldh* F | CTTCCTCGTTGCTGCTAACC | qRT-PCR |
| *ldh* R | AGCAAGTGCTTGACGGAAAC | qRT-PCR |
