# Supplementary material for: Acetylation of Lactate Dehydrogenase Negatively Regulates the Acidogenicity of Streptococcus mutans
Source: mBio. 2022 Aug 31;13(5):e02013-22. doi: 10.1128/mbio.02013-22 (PMC9600946; doi:10.1128/mbio.02013-22)
Supplement: TABLE S4 [file mbio.02013-22-s0009.docx]

**TABLE S4** Bacterial strains and plasmids used in this study.

| **Strains or plasmids** | **Description** | **Source** |
| --- | --- | --- |
| ***S. mutans*** |  |  |
| UA159 | Wild type strain | ATCC 700610 |
| UA159/pDL278 | UA159/pDL278; Spe^r^ | This study |
| UA159/pDL278*-actA* | UA159/pDL278*-386; Spe^r^* | This study |
| UA159/pDL278*-actB* | UA159/pDL278*-639; Spe^r^* | This study |
| UA159/pDL278*-actC* | UA159/pDL278*-844; Spe^r^* | This study |
| UA159/pDL278*-actD* | UA159/pDL278*-850; Spe^r^* | This study |
| UA159/pDL278*-actE* | UA159/pDL278*-1072c; Spe^r^* | This study |
| UA159/pDL278*-actF* | UA159/pDL278*-1154c; Spe^r^* | This study |
| UA159/pDL278*-actG* | UA159/pDL278*-1253c; Spe^r^* | This study |
| UA159/pDL278*-actH* | UA159/pDL278*-1392c; Spe^r^* | This study |
| UA159/pDL278*-actI* | UA159/pDL278*-1483c; Spe^r^* | This study |
| UA159/pDL278*-actJ* | UA159/pDL278*-1511c; Spe^r^* | This study |
| UA159/pDL278*-actK* | UA159/pDL278*-1558c; Spe^r^* | This study |
| UA159/pDL278*-actL* | UA159/pDL278*-1654c; Spe^r^* | This study |
| UA159/pDL278*-actM* | UA159/pDL278*-1730c; Spe^r^* | This study |
| UA159/pDL278*-actN* | UA159/pDL278*-2055; Spe^r^* | This study |
| UA159/pDL278*-actO* | UA159/pDL278*-2072c; Spe^r^* | This study |
| UA159 Δ*actA* | UA159 Δ*386*; Em^s^; p-Cl-Phe^r^ | This study |
| ***E. coli*** |  |  |
| DH5α | F- φ80dlacZΔM15 Δ(lacZYA-argF)U169 deoR recA1 endA1 hsdR17 | Laboratory stock |
|  | (rk-，mk+) phoA supE44 λ- thi-1 gyrA96 relA1 |  |
| BL21(DE3) | F- ompT hsdS B(rB-mB-)dcm gal (DE3) | Novagen |
| **Plasmids** |  |  |
| pET28a | Kan^r^ expression vector with the 6His-tag coding sequence | Novagen |
| pETadcR | pET derivative for expression 6His-StsR | This study |
| pDL278 | E. coli-Streptococcus shuttle vector (Spe^r^) | LeBlanc et al., 1992 |
| pDL278*-actA* | pDL278 derivative for overexpression of *smu_386* in *S. mutans* | This study |
| pDL278*-actB* | pDL278 derivative for overexpression of *smu_639* in *S. mutans* | This study |
| pDL278*-actC* | pDL278 derivative for overexpression of *smu_844* in *S. mutans* | This study |
| pDL278*-actD* | pDL278 derivative for overexpression of *smu_850* in *S. mutans* | This study |
| pDL278*-actE* | pDL278 derivative for overexpression of *smu_1072c* in *S. mutans* | This study |
| pDL278*-actF* | pDL278 derivative for overexpression of *smu_1154c* in *S. mutans* | This study |
| pDL278*-actG* | pDL278 derivative for overexpression of *smu_1253c* in *S. mutans* | This study |
| pDL278*-actH* | pDL278 derivative for overexpression of *smu_1392c* in *S. mutans* | This study |
| pDL278*-actI* | pDL278 derivative for overexpression of *smu_1483c* in *S. mutans* | This study |
| pDL278*-actJ* | pDL278 derivative for overexpression of *smu_1511c* in *S. mutans* | This study |
| pDL278*-actK* | pDL278 derivative for overexpression of *smu_1558c* in *S. mutans* | This study |
| pDL278*-actL* | pDL278 derivative for overexpression of *smu_1654c* in *S. mutans* | This study |
| pDL278*-actM* | pDL278 derivative for overexpression of *smu_1730c* in *S. mutans* | This study |
| pDL278*-actN* | pDL278 derivative for overexpression of *smu_2055* in *S. mutans* | This study |
| pDL278*-actO* | pDL278 derivative for overexpression of *smu_2072c* in *S. mutans* | This study |
